# Outcomes Following Colorectal Cancer Surgeries at the Basildon and Thurrock University Hospital

**DOI:** 10.7759/cureus.61261

**Published:** 2024-05-28

**Authors:** Omotara Kafayat Lesi, Ebuwa Igho-Osagie, Nida Bashir, Shashi Kumar, Spencer Probert, Methusha Sakthipakan, Lipsos Constantino, Suvarna Paratharajan, Suliman Ahmad, Samer-ul Haque

**Affiliations:** 1 General and Colorectal Surgery, Basildon and Thurrock University Hospital, Basildon, GBR; 2 Medicine, Merck & Co., New York, USA; 3 Surgery, Basildon and Thurrock University Hospital, Basildon, GBR; 4 General Surgery, Basildon and Thurrock University Hospital, Basildon, GBR

**Keywords:** length of hospital stay, morbidity and mortality, post-operative complications, surgical outcomes, colorectal cancer

## Abstract

Aim

We reviewed surgical outcomes for patients with colorectal cancer resections in Basildon and Thurrock University Hospital between April 2019 and March 2020.

Methods

Clinical characteristics of 141 patients who underwent surgical resection for colorectal cancer at the district hospital were assessed and reported, including tumor site, disease stage, and type of surgical resection performed. We reviewed 30- and 90-day postoperative mortality, postoperative complications, return to the theater, and extended hospital stay data for these patients. The results of our review across measured outcomes were compared to the national average from the National Bowel Cancer Audit (NBOCA) Report.

Results

Clinical data and health outcomes for 141 patients with colorectal cancer resections within the index year were reviewed. The mean age at diagnosis was 68.9 (12.5) years. Among the patients, 61 (43.3%) were female, and 59 (41.8%) had Stage III and IV colorectal cancer. Around 95 (67.4%) had the colon as the primary tumor site, while 46 (32.6%) had the primary tumor site in the rectum. Of the patients, 17 (12.1%) had emergency surgeries, and 124 (87.9%) underwent laparoscopic surgery. Right hemicolectomy was the most common operation performed in 58 patients (41.1%). The average length of stay was 7.8 (6.6) days; the length of stay was similar for both colonic and rectal resections. Low 30-day and 90-day mortality rates of (1/141) 0.71% and (2/141) 1.4%, respectively, were observed compared to the 90-day United Kingdom (UK) national average mortality rate of 2.7% in 2019/20. Around 30 (21.3%) of the patients developed postoperative complications within 30 days of surgery. Only six out of 30 postoperative complications were classified as Clavien-Dindo Grade III.

Conclusion

Surgical outcomes for patients with colorectal cancer in our district general hospital are similar to or lower than the national averages estimated by NBOCA. To further strengthen surgical care delivery and improve patient outcomes in the United Kingdom, there is a need to improve surgical techniques and quality improvement processes.

## Introduction

Colorectal cancer (CRC) is the fourth most common cancer [1] and the second leading cause of cancer death in the United Kingdom (UK) and worldwide [2,3,4]. Forty-two thousand new cases of CRC are diagnosed annually in the UK [5], and this results in an 11% incidence rate of all cancers [1,6,7]. Incidence rates of colorectal cancer have remained stable in the UK since the 1990s and are projected to fall by 8% between 2025 and 2040 [1]. Surgery is the mainstay of treatment for colorectal cancer [8], with chemotherapy and radiotherapy often used as neo-adjuvant or adjuvant therapies. [9] High disease-free survival rates are observed with surgery in earlier disease stages [10], but colorectal cancer surgery is still associated with considerable perioperative morbidity and mortality [11]. Between 1998 and 2006, the 30-day postoperative mortality rate was estimated to be as high as 7% across National Health Services (NHS) hospitals in England [11]. This mortality rate was highest in elderly males, patients with socioeconomic deprivation, late-stage disease, multi-morbidity, and among those who underwent emergency surgery [12].

Several surgical innovations and perioperative measures have been adopted by healthcare stakeholders over time to improve surgical outcomes for patients with CRC [13]. The use of laparoscopic and laparoscopic-assisted surgeries has reduced patients’ length of hospital stay and facilitated a quicker return to normal daily activities after surgery [13]. Enhanced recovery after surgery, a multimodal approach that optimizes patients’ physiological and psychological outcomes across the preoperative, intraoperative, and postoperative phases of care, has also been used to reduce postoperative complications and length of stay for patients after surgery [14].

In the UK, clinical audits review surgical practices and procedures against gold standards and ensure that surgeons and other caregivers adhere to best practices and standards [15]. The use of audits has led to improvements in overall CRC care and its associated health outcomes. The most recent (2022) National Bowel Cancer Audit (NBOCA) [15] reports a reduction in unplanned returns to the theater (8.3% to 7.2% between 2016 and 2021), with a further decrease in postoperative hospital stays after elective surgeries. However, there has been a minimal increase in 90-day postoperative mortality (2.7% to 3.1% between 2019 and 2021) [15].

Studies have shown mixed associations between CRC surgical volume in a hospital system and CRC-related health outcomes [16-18]. Several studies have demonstrated that higher surgical volume is linked with a reduction in post-surgical mortality [19-21]. In contrast, other studies [17,22] have not shown clinically significant differences in operative mortality and long-term survival among hospital groups in patients who had rectal cancer resections.

Basildon and Thurrock University Hospital (BTUH) is a district general hospital that provides health care services to 405,000 people residing in South Essex, United Kingdom. It is an average-volume CRC surgical care center with a colorectal surgical unit consisting of colorectal consultants, middle-grade doctors, junior doctors, colorectal specialist nurses, physiotherapists, occupational therapists, and a nutrition team.

## Materials and methods

Study design

This study was a single-center, retrospective review of 141 patients who underwent major colorectal cancer resections between April 2019 and March 2020 at Basildon and Thurrock University Hospital (BTUH), Basildon, United Kingdom. Major resection was defined as right hemicolectomy, left hemicolectomy, segmental resection, sigmoid colectomy, Hartmann’s procedure, subtotal colectomy, anterior resection, and abdominoperineal resection.

We compared the results with those observed nationally and in other hospital centers. In addition, we reviewed morbidity, mortality, and complications associated with CRC surgeries and examined exploratory associations between different risk factors and various clinical outcomes.

We also reviewed several outcomes of interest, including 30- and 90-day mortality, postoperative complications, return to the theater, and extended hospital stays. 

Return to the theater was defined as a postoperative procedural intervention between one and 30 days after the index surgery, while postoperative complications were defined as complications occurring within 30 days of surgery and graded using the Clavien-Dindo system.

Participants

Study participants were both elective and emergency adults over 18 years old with colorectal cancers. Elective patients had a confirmed histological diagnosis of colorectal cancer through colonoscopy or flexible sigmoidoscopy. They had been discussed at the colorectal multidisciplinary team meeting for curative intent with surgery and/or chemoradiotherapy (neo-adjuvant/adjuvant). Patients for best supportive care and palliative care were excluded from the study. Emergency patients were those who presented with obstruction or perforation and were newly diagnosed with colorectal cancer or were awaiting elective surgery. 

Study variables

Demographic, behavioral, and clinical variables were extracted from the clinical records of the study participants. Demographic variables included age, gender, body mass index, comorbidities, the American Society of Anesthesiologists (ASA) score, performance score, smoking, and alcohol status. 

Comorbidity included any comorbid condition one month before and up to 12 months after surgery. The ASA physical status classification was used for assessing physical fitness, and the World Health Organization (WHO) classification was used to categorize the performance status of the patients before surgery.

Clinical variables were tumor, node, metastases (TNM) or American Joint Committee on Cancer (AJCC) stage of cancer, location of the tumor, type of surgery done, and presence or absence of a stoma. Patients with caecal, ascending colon, hepatic flexure, transverse colon, splenic flexure, descending colon, and sigmoid colon cancers were grouped as colon cancers, while those with recto-sigmoid and rectal cancers were grouped as rectal cancers. The type of surgical procedure done was documented as either open or laparoscopic surgery, and the conversion rate from laparoscopic to open surgery was documented. The final TNM/AJCC stage was derived after a pathologic review of resected specimens.

## Results

Patient population: demographics and clinical characteristics

One hundred and forty-one (141) patients had colorectal cancer surgery between April 2019 and March 2020 at Basildon and Thurrock University Hospital, United Kingdom. Around 61 (43.3%) were female, and the mean age at diagnosis was 68.9 (12.5) years. Twenty-four (17%) of the patients were under 60 years old, and 29 (20.6%) were 80 years or older. The mean body mass index (BMI) was 27.7 (5.1), with 41 (29.1%) of patients being obese at presentation. Only 22 (15.6%) of patients were current smokers.

Patients had an average of 3.3 (SD 1.7) co-morbidities. The proportion of patients with three or more co-morbidities was 82 (58.2%). Only one patient had no identified co-morbidity. Hypertension was the most common (54, 38.3%), followed by atrial fibrillation (18, 12.8%), type 2 diabetes mellitus (15, 10.6%), and chronic obstructive pulmonary disease (8, 5.7%). Around 82 (58.2%) had early-stage disease (Stage I and II), with 95 (67.4%) having their primary tumor site in the colon. Of the operated patients, 70 (49.6%) had an ASA score II, while 54 (38.3%) had an ASA score III. Seventeen (12.1%) of patients were operated on as emergencies, and 124 (87.9%) underwent laparoscopic surgery.

Outcomes

Of the patients, 58 (41.1%) had right hemicolectomies, while 50 (35.5%) had anterior resections. Segmental resection 1 (0.7%) and Hartman’s procedure 2 (1.4%) were the least performed surgeries among patients operated upon in the hospital. Emergency resections accounted for 17 (12.1%) of the surgeries conducted. The most frequent reason for emergency resections was bowel obstruction. Twenty (14.2%) of the patients who had primary resections also had permanent colostomies made. These included Hartman's procedure (4, 2.8%), left hemicolectomy (2, 1.4%), anterior resections (9, 6.4%), and abdominoperineal excision (5, 3.5%). Only nine patients (6.4%) had neo-adjuvant chemoradiotherapy/chemotherapy before surgery, with only one patient requiring a defunctioning loop colostomy before commencing neo-adjuvant treatment (Table 1).

**Table 1:** Clinical and demographic characteristics of patients with colorectal cancer in Basildon Hospital Trust between 2019 and 2020 (n=141) AJCC: American Joint Committee on Cancer; TNM: tumor, node, metastasis

Variable	N(%)
Mean age (years)	68.9 (12.5) - Mean (SD)
Age categories (years)	
<60	24(17%)
60-69	38(27%)
70-79	50(35.5%)
≥80	29(20.6%)
Sex	
Female	61(43.3%)
Male	80 (56.7%)
American Society of Anesthesiologists (ASA) score	
1	12 (8.5%)
2	70 (49.6%)
3	54 (38.3%)
4	4 (2.8%)
5	1 (0.7%)
World Health Organization (WHO) performance score	
0	93 (66%)
1	38 (27%)
2	9 (6.4%)
3	1 (0.7%)
Mean body mass index (BMI)	27.7 (5.1) - Mean (SD)
BMI category	
Underweight	4 (2.8%)
Normal	41(29.1%)
Overweight	46(32.6%)
Obese	41(29.1%)
Smoking status	
Ex-smoker	24 (17%)
No	95 (67.4%)
Yes	22 (15.6%)
Alcohol intake	
No	80 (56.7%)
Yes	61 (43.3%)
Comorbidities (mean)	3.3 (1.7) - Mean (SD)
Number of comorbidities	
0	22 (15.6%)
1	37 (26.2%)
>2	82 (58.2%)
Tumor primary site	
Colon	95 (67.4%)
Rectum	46 (32.6%)
Type of surgery	
Elective	124 (87.9%)
Emergency	17 (12.1%)
Surgical access	
Laparoscopy	124 (87.9%)
Open	17 (12.1%)
Conversion	28 (19.9%)
(AJCC/TNM)* Tumour stage (final)	
Early (Stage I & II)	82 (58.2%)
Late (Stage III & IV)	59 (41.8%)
Surgical procedures	
Right hemicolectomy	58 (41.1%)
Left hemicolectomy	7 (5%)
Segmental resection	1 (0.7%)
Sigmoid colectomy	8 (5.7%)
Hartman’s procedure	4 (2.8%)
Subtotal colectomy	8 (5.7%)
Anterior resection	50 (35.5%)
Abdominoperineal resection (APER)	5 (3.5%)
Stoma	
Ileostomy	30 (21.3%)
Colostomy	20 (14.2%)
Double barrel	2 (1.4%)
No stoma	89 (63.1%)

The mean length of hospital admission stay after surgery was 7.8 days (6.6); the median was six days (interquartile range (IQR): 4,9 days). There was no difference in the length of hospital stay between colonic and rectal resections. Around 55 (39%) of patients had a post-surgery hospital stay greater than seven days, and 19 (13.5%) had a post-surgery length of stay greater than 14 days.

30-day and 90-day mortality

Only one patient (1/141) died within 30 days of surgical resection. Two other patients died within 90 days post-surgery.

Return to theatre, emergency readmission, and extended hospital stay

Within 30 days after surgery, 30 (21.3%) of the patients developed postoperative complications. Only six out of the 30 patients who developed postoperative complications required a return to theater or interventional radiology (Clavien-Dindo classification: Grade III). The common postoperative complications were chest infections (four), anastomotic leaks (three), and wound infections (three). Five patients required a return to the theater due to an anastomotic leak or intra-abdominal sepsis. Nine patients had unplanned re-admission to the hospital within 30 days of surgery due to cardiac arrhythmia, intra-abdominal collection, wound infection, or anastomotic leak. About half of them required only medical management during readmission (Table 2).

**Table 2:** Postoperative complications (n=30)

Postoperative complications	n (%)	Clavien-Dindo Grade	Unplanned re-admission n(%)
Wound infections	3 (10%)	II	2 (6.7%)
Anastomotic leak	3 (10%)	III b (two patients), II (one patient)	2 (6.7%)
Neutropenic sepsis	1 (3.3%)	II	-
Intra-abdominal sepsis/collection	2 (6.7%)	III a, III b	1 (3.3%)
Sepsis	3 (10%)	II	-
Chest infection	4 (13.3%)	II	-
Bowel ileus	1 (3.3%)	II	-
Type 2 myocardial infarction	1 (3.3%)	II	-
Acute kidney injury	1 (3.3%)	II	1 (3.3%)
High stoma output	3 (10%)	II	1 (3.3%)
Ischaemic stoma	1 (3.3%)	III b	-
Rehabilitation bed	1 (3.3%)	I	-
Hypotension	1 (3.3%)	IV	-
Anaemia	1 (3.3%)	II	-
Urinary tract infection	1 (3.3%)	II	-
Ischaemic colon	1 (3.3%)	III b	-
Atrial flutter	1 (3.3%)	II	1 (3.3%)
Internal jugular vein thrombus	1 (3.3%)	II	1 (3.3%)

## Discussion

In this study, we reviewed the surgical outcomes of colorectal cancer patients in Basildon and Thurrock Hospital who received surgical resections as their primary colorectal cancer treatment. We observed low 30- and 90-day mortality rates (1/141; 0.71%; 2/141; 1.4%, respectively) as well as low return-to-theatre rates (5, 3.5%), following post-surgical complications.

Several studies examining 30-day mortality in surgically treated patients with colorectal cancer have demonstrated much higher mortality rates than those observed in our center [12,23-25]. Reviewing CRC patients who underwent colorectal resections between 1998 and 2006, Morris et al. (2011) found 30-day post-surgical resection mortality to be 6.7% [21]. A more recent study by Mik et al. (2016) in Poland reported a 30-day mortality rate of 3.5% [24]. Other studies by Frederiksen and Widdison found similar 30-day mortality rates of 4.8% and 4%, respectively [23,25]. It appears that 30-day mortality rates following colorectal cancer surgery have fallen over time in the UK and across Europe [15]. The UK National Cancer Registration and Analysis Service (NCRAS) notes that 30-day postoperative mortality fell from 6.9% to 5.8% over nine years across England hospitals [26].

The 90-day mortality rate observed in this study was similar to those observed in other studies [15,27]. Hureibi et al.’s [27] study of colorectal resections in England between 2010 and 2015 showed a 90-day mortality rate of 2.6% (vs. 1.4% in our study) [27], and the 2022 NBOCA Annual Audit reported an overall 90-day postoperative mortality of 3.1% across all UK hospitals. Our study reports a much lower 90-day mortality rate than the national average [15].

In total, three patients died between 30 and 90 days post-surgery. These patients were elderly, had multiple co-morbidities, and two out of three of them had emergency colectomies and stoma formation as part of their cancer treatment. These risk factors of elderly age and emergency surgery are independently associated with 30- and 90-day mortality in colorectal cancer patients undergoing surgical resection [8,28,29] The NCRAS notes that mortality is still high in the elderly, among males, the socioeconomically deprived, people with advanced stages of disease, and those who had emergency surgeries [26], despite an overall drop in mortality rates in surgically treated CRC patients.

Return to theater is linked to prolonged hospital stays and increased mortality in CRC patients [30]. Return to theatre was seen in elective patients, with none occurring for emergency patients, and the reasons were anastomotic leak, intra-abdominal sepsis, and stoma complications; these are common post-surgical complications associated with return to theatre after colorectal surgeries [31,32]. Several studies have shown that lower return to theater rates may be due to a higher proportion of right-sided resections than left resections [31,31,33]. However, this study showed similar proportions of left-sided vs. right-sided resections.

Patients in our center who experienced an extended length of hospital stay after surgery, defined as fourteen or more days on admission post-surgery, were more likely to have left-sided resections (anterior resections, left hemicolectomies), open surgeries, as well as stoma formation. Extended hospital stays after CRC surgical resections are associated with open techniques, emergency surgeries, older patients, and left-sided resections [34,35] Over time, in the UK, there has been a decrease in the length of post-surgical hospital admissions for patients undergoing CRC surgery [36]. In our center, the median length of stay in our study was six days (IQR: 4,9), similar to the six-day average length of hospital stay reported in the 2022 NBCOA report for all hospitals in the UK.

Our study has several strengths: we examined patient outcomes in a single NHS trust and compared them with established averages from the UK’s official hospital outcomes auditing organization. In this regard, we could easily identify potential areas for improvement in service delivery for patients receiving care from this trust. Also, only a few studies in the United Kingdom have examined surgical outcomes in patients receiving treatments for colorectal cancer in recent years. This study adds to and updates evidence on health outcomes for CRC patients in the UK receiving surgery.

The study has a few limitations. Our sample size was too small to allow for robust multivariable estimations of the predictors of observed outcomes. However, the low rate of negative outcomes within 30 and 90 days may preclude such analyses, especially as cancer treatments improve over time. As this is a low-volume center where most patients with locally advanced disease are referred to tertiary centers, this may account for its low incidence of morbidity and mortality. Future studies seeking to understand predictors of negative health outcomes in this population may require merging hospital records across multiple NHS trusts and hospitals.

## Conclusions

In this study, we found that health outcomes in patients with colorectal cancer in a medium-sized district general hospital in the United Kingdom, including 30-day and 90-day postoperative mortality, were similar or lower than the national averages. The results of this study have implications for practice within the NHS. Individual NHS trusts may conduct similar reviews, comparing their outcomes with local, regional, or national averages. This may help hospitals identify areas for improvement in the care of patients with colorectal cancer, thereby improving the quality of service delivery across the country.
